# Skeletal Muscle Atrophy in Simulated Microgravity Might Be Triggered by Immune-Related microRNAs

**DOI:** 10.3389/fphys.2018.01926

**Published:** 2019-01-10

**Authors:** Laura Teodori, Alessandra Costa, Luigi Campanella, Maria C. Albertini

**Affiliations:** ^1^Diagnostic and Metrology Laboratory, TECFIS-FSN, ENEA, Frascati, Italy; ^2^Department of Chemistry, Sapienza University of Rome, Rome, Italy; ^3^Department of Biomolecular Sciences, University of Urbino Carlo Bo, Urbino, Italy

**Keywords:** space flight, bioinformatics, miRs prediction, web-based platform, immune function deregulation, skeletal muscle atrophy

## Abstract

Exposure to microgravity induces skeletal muscle disorders including atrophy, muscle force decrease, fiber-type shift. Microgravity also contributes to immune-function alterations and modifies microRNAs (miRs) expression. To understand the link between microgravity-induced skeletal muscle atrophy and immune function deregulation, a bioinformatics study was performed. The web platform MiRNet was used for miRs-targets interaction analysis from previous proteomic studies on human *soleus* (SOL) and *vastus lateralis* (VL) muscles. We predicted miRs targeting deregulated gene expression following bed rest as a model of microgravity exposure; namely, let-7a-5p, miR-125b-5p for over-expressed genes in SOL and VL; miR-1-3p, miR-125b-5p and miR-1-3p, miR-95-5p for down-expressed genes in VL and SOL. The predicted miRs have important immune functions, exhibiting a significant role on both inflammation and atrophy. Let-7a down-expression leads to proliferation pathways promotion and differentiation pathway inhibition, whereas miR-1-3p over-expression yields anti-proliferative effect, promoting early differentiation. Such conflicting signals could lead to impairment between proliferation and differentiation in skeletal muscles. Moreover, promotion of an M2-like macrophage phenotype (IL-13, IL-10) by let-7a down-regulation and simultaneous promotion of an M1-like macrophage (IL-6, TNF-α) phenotype through the over-expression of EEF2 lead to a deregulation between M1/M2 tuning, that is responsible for a first pro-inflammatory/proliferative phase followed by an anti-inflammatory pro-myogenic phase during skeletal muscle regeneration after injury. These observations are important to understand the mechanism by which inflammation may play a significant role in skeletal muscle dysfunction in spaceflights, providing new links between immune response and skeletal muscle deregulation, which may be useful to further investigate possible therapeutic intervention.

## Introduction

Microgravity poses one of the greatest risks to astronauts during prolonged missions (Williams et al., 2009). Exposure to microgravity results in decreased muscle strength and endurance, raising concerns that muscle atrophy could increase long-term spaceflight risk unless adequate countermeasures were undertaken. Indeed, the employed physical exercise countermeasures are still incapable of preventing muscle atrophy, thus increasing needs for development of more focused program. Immune deregulation is also a major issue during spaceflights and was first observed in astronauts following missions, making them most exposed to infections (Johnston et al., 1975). Muscle atrophy is a feature of many diseases affecting skeletal muscle and it also responsible for muscle disease exacerbation (Kovanda et al., 2018). Exposure to microgravity during spaceflights might modify miRs expression as compared to normal gravity. MiRs are short non-coding RNAs able to regulate gene expression. Muscle specific miRs (myomiRs) regulate several processes in skeletal muscle, such as myogenesis, muscle homeostasis and response to external stimuli. Muscle-specific miRs (myomiRs) control various processes in skeletal muscles, from myogenesis and muscle homeostasis to responses to environmental stimuli, such as exercise. MiRs are critical regulators of both adult skeletal muscle differentiation/maintenance and inflammatory cytokine signaling (Bartel, 2004; Baltimore et al., 2008; Sonkoly et al., 2008). We hypothesized that links might exist between skeletal muscle atrophy and inflammation deregulation triggered by microgravity. The aim of the present work is to explore the mechanism by which the immune response to simulated microgravity may play a role in skeletal muscle dysfunction in spaceflights, providing a new mechanism linking the action of inflammatory cytokines to skeletal muscle deregulation. Deregulated proteins associated to bed rest as a microgravity model from a previous study (Moriggi et al., 2010) were employed to build a network identifying new putative miRs involved. MiRNet, an easy-to-use web-based tool^1^, helped us to identify new regulatory mechanisms associated to immune microgravity muscle atrophy.

## Materials and Methods

The proteome profile previously performed on muscle biopsies (from VL to SOL) from 12 healthy subjects before and after 55 days of bed rest (Moriggi et al., 2010) has been used for this study. The proteins that showed a differential expression in the two conditions in VL and SOL muscles have been divided into 2 different groups: over- and down-expressed. The Swiss-Prot accession number of the deregulated proteins (genes) was used in PubMed^2^ to look for the associated official gene symbol. This latter has been used in miRNet to build a network identifying new putative miRs-targets interactions. The network shows the interactions existing between a miRNA and the genes modulated^1^. We used the mRNA area and we uploaded 4 different lists of gene symbols: VL over-expressed; VL down-expressed; SOL over-expressed; SOL down-expressed. We first performed the analysis inserting *H. sapiens* as organism and the official gene symbol as ID type. We also repeated a second analysis choosing “muscle” as tissue (human only) option. In this way, a network with nodes and connections between our genes and miRs has been built.

## Results

We used the proteome profile from Moriggi et al. (2010) where 55 days bed rest modulated different muscle genes. A list of modulated SOL and VL miRs was used for miRNet analysis to look for miRs-targets connections. We found putative miRs associated to up- and down-regulated genes (Table 1). Furthermore, when the analysis was performed choosing mRNAs from human muscle, different miRs emerged.

**Table 1:** Results obtained by miRNet analysis.

Deregulated proteins	microRNAs (gene symbols; gene IDs)
VL Over-expressed	miR-484
	miR-92a-3p
	miR-16-5p
	miR-615-3p
	miR-222-3p
	miR-320a
**VL Over-expressed in muscle**	**let-7a-5p (EEF2; ID: 1938; eukaryotic translation elongation factor 2) (VCL; ID: 7414; vinculin)**
	**miR-125b-5p (VDAC1; ID: 7416; voltage dependent anion channel 1) (VCL; ID: 7414; vinculin)**
VL Down-expressed	miR-5196-5p
	miR-4747-5p
**VL Down-expressed in muscle**	**miR-1-3p (SUCLA2; ID: 8803; succinate-CoA ligase ADP-forming beta subunit) (PRDX2; ID: 7001; peroxiredoxin 2)**
	**miR-125b-5p (PRDX2; ID: 7001; peroxiredoxin 2)**
SOL Over-expressed	miR-222-3p
	miR-let-7b-5p
	miR-34a-5p
	miR-320a
**SOL Over-expressed in muscle**	**let-7a-5p (VCL; ID: 7414; vinculin) miR-125b-5p (VCL; ID: 7414; vinculin)**
SOL Down-expressed	miR-30c-5p
	miR-1-3p
	miR-30a-5p
**SOL Down-expressed in muscle**	**miR-1-3p (SUCLA2; ID: 8803; succinate-CoA ligase ADP-forming beta subunit) (ACTC1; ID: 70; actin, alpha, cardiac muscle 1) miR-95-5p (ACTC1; ID: 70; actin, alpha, cardiac muscle 1)**

*We reported the putative microRNAs that are associated to the over- and down-expressed VL/SOL proteins (genes). The genes associated to muscle tissue are also reported in parenthesis (bold). VL, vastus lateralis human muscle; SOL, human soleus.*

**FIGURE 1:**
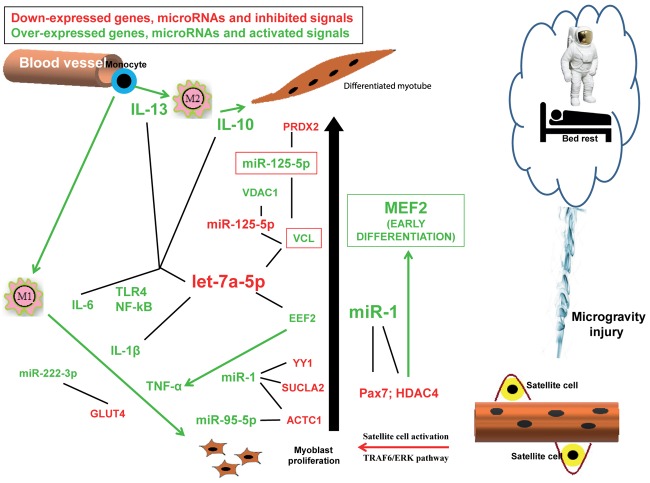
Hypothesis of the conflicting signals responsible for atrophy observed during reduced gravity environment. Down-expressed genes, microRNAs and inhibited signals (arrows) are indicated in red, while over-expressed genes, microRNAs and activated signals (arrows) are indicated in green. Black lines indicate the possible interactions between mRNA and microRNAs evidenced by the miRNet analysis.

We performed a bibliographic analysis of the muscle miRs and their putative targets emerged from the miRNet analysis. We found that let-7a-5p and miR-1 are evidently involved in a proliferation/differentiation conflicting signaling (Figure 1) and their down- and up-regulation (respectively) play a fundamental role in tissue adaptation under simulated microgravity, resulting in skeletal muscle atrophy, strictly associated to immune-function dysregulation.

## Discussion

The results of our bioinformatic analysis predicted some miRs dysregulation in healthy subjects undergoing bed rest as microgravity model. Our results evidenced that let-7 down-regulation and simultaneous miR-1 up-regulation might play a fundamental role in tissue adaptation under microgravity resulting in skeletal muscle atrophy. Let-7 promotes cell differentiation and cellular proliferation inhibition in several cellular systems (Roush and Slack, 2008). The let-7miRs family plays a key role in modulating inflammatory responses as well (Song and Lee, 2015). Let-7a is involved in inflammation processes by participating to NF-κB and angiogenesis signaling (Song et al., 2016). When miRs of let-7 family are downregulated, the levels of IL-6 and IL-10 cytokines are downregulated too (Liu et al., 2011; Schulte et al., 2011). Moreover decreased levels of let-7 following microbial infection enhance the expression of TLR4 (Androulidaki et al., 2009; Hu et al., 2009). Let-t is also related to adaptive immune response since it has been show a regulation by let-7 of the production of IL-13 by T lymphocyte during the inflammatory process due to airway allergic response. Indeed, let-7 can inhibit the anti-inflammatory cytokine IL-13 secretion (Fan et al., 2016); on the other hand let-7 miRs expression is dynamically regulated in response to TNF-α (and other factors as serum glucose). Thus let-7 down-expression, as in simulated microgravity, exacerbates inflammation. Finally, our bioinformatics (*in silico*) analysis of the interactions between miRs and the pathways involved in the inflammatory process, and in skeletal muscle wasting, such as TNF-alpha and NF-kB pathways identified let-7 family as one of the main predicted regulators of the aforementioned pathways. Noteworthy, many transmembrane receptors involved in these pathways (TNFR1, TNFR2, and IL1R) are targets of let-7 miRNA (Brennan et al., 2017).

Our *in silico* analysis showed that let-7 targets vinculin (VC), a protein connecting integrins to actin filaments, and that is recruited to focal adhesions (FAs) in response to force. VCL is suggested to be linked to FA mechanosensitivity and it is essential for FA stabilization under force (King et al., 2018). Migration of satellite cells (SC), necessary for skeletal muscle development and regeneration, is strictly dependant on the formation of mature FA connecting the cell to the extracellular matrix (Goetsch et al., 2014). In particular VCL is critical during the remodeling of the junction, due to the force exerted by the cytoskeleton, to avoid junction opening (Huveneers et al., 2012). TNF-α induces substantial reorganization of actin cytoskeleton and FA (Koukouritaki et al., 1999). In 55 days bed rest, VCL might fail to ensure proper migration and alignment for the cell to differentiate. Cells expressing VCL by 20% over endogenous level had altered locomotory properties. VCL is a key control protein for FA modeling and it is regulated by tension (Spanjaard and de Rooij, 2013). VCL expression at FA is negatively regulated by TNF-alpha that also indices cytoskeletal actin depolymerization (Scott and Panitch, 2014). Let-7 also targets EEF2 and, in turn, EEF2 suppresses let-7 expression (Ding et al., 2008). EEF2 activation activates subsequent TNF-α elongation (via MKK3/6-p38γ/δ pathway).

The restoration of let-7 levels might represent an interesting strategy for therapeutic approaches to prevent up-regulation of key proteins implicated in microgravity-driven inflammation and other key pathological hallmarks of atrophy processes.

Our *in silico* research also predicted miR-1-3p deregulation. MiR-1 family (belonging to myomiRs) controls myosin content, fiber type and muscle performance and directly targets HDAC4 (histone deacetylase 4) (Zuo et al., 2015). HDAC4 is directly inhibited by miR-1. This inhibition is responsible for cell-cycle arrest in G1 and G2, an anti-proliferative effect typical of HDAC inhibitors (Sun et al., 2015). HDAC4 has an inhibitory effects upon MEF2 (myocyte enhancer factor) transcription factor, responsible for the activation of early myogenic differentiation (Sun et al., 2015). A mutual regulation also exists between mir-1 and YY1, an inhibitor of myogenic differentiation; in fact miR-1 binds the 3′-UTR of YY1 and YY1 targets miR-1 (Sun et al., 2015).

Expression of miR-1 is regulated by nNOS signaling through HDCA2. HDAC may regulate the expression of iNos and NF-kB dependent genes, which can further induce the expression of cytokines (Kharitonov and Ito, 2009). MiR-1 also controls G6PD, a relevant enzyme in oxidative stress response (Sun et al., 2015).

Glucocorticoids induce skeletal muscle atrophy with miR-1 expression through glucocorticoid receptor and myostatin. The results suggest that miR-1 is a catabolic miR and might have a central role in microgravity-induced skeletal muscle atrophy. MiR-1 also blocks Pax7 which has been suggested to have a role in the maintenance of the proliferative phase through the prevention of early differentiation, thus miR-1 up-regulation results in a block of proliferation and early differentiation induction. Pax7 is also a regulator of MyoD and may be involved in MyoD up-regulation observed during SC activation. Pax7 has also been demonstrated to be sufficient for the activation of the myogenic program in CD45+/Sca1+ cells isolated from adult skeletal muscle tissue (Zammit et al., 2006). Hosoyama et al. (2017) hypothesize that human skeletal muscle stem/progenitor cells pool may be reduced during microgravity when Pax7 is down-regulated through TRAF6/ERK signaling pathway.

Our results also showed that miR-125b-5p is associated either to over- and down-expressed muscle VL genes and associated to over-expressed muscle SOL VCL gene. MiR-125b-5p targets mitochondrial apoptotic pathways and regulates the phenotype of macrophages targeting B7-H4 (Diao et al., 2017). MiR-125b-5p over-expression down-regulates B7-H4 expression in macrophage. B7-H4, an immunoglobulin superfamily molecule that has been shown to inhibit T cell responses through cell cycle arrest and inhibition of T cell proliferation and cytokine release. B7-H4 has been also identified in skeletal muscle. MiR-125b plays a role in innate immune response. MiR-125-b induces fibrosis by targeting apelin (Chen et al., 2017). MiR-125a and miR-125b indirectly activate NK-kB pathway via suppression of TNF-alpha-induced protein 3 (TNFAIP3, A20) (Chen et al., 2017). Down-regulation of miR-125b-5p increases inflammation (Chen et al., 2017).

Our results also predicted that miR-95-5p is associated with ACTC1 down-expressed SOL gene muscle. MiR-95-5p has been proposed as a new miR biomarker candidate in muscular dystrophy (dystromir). MiR-95 promotes myogenic differentiation by down-regulating the AIMP2 translation. ACTC1 is an AIMP2 interacting protein and both have been found to be implicated in FA.

The results also demonstrated an over-expression of miR-222-3p and miR-320a in both VL and SL. SLC2A4 gene (GLUT4 protein) (Xu et al., 2015) is a direct target of miR-222-3p. Limb immobilization halved muscle GLUT4 protein concentration in animal models. GLUT4 decrease is also associated with insulin resistance and high levels of inflammatory markers. The oxidative stress-responsive microR-320a is a skeletal muscle mitochondrial metabolism and migration regulator. It regulates glycolysis by directly downregulating phosphofructokinase, in diverse biological systems (Tang et al., 2012). Increased levels of miR-320a may account for an impairment of the glycolytic system by suppressing a key enzyme in the glycolysis process.

Several are the miRs associated to skeletal muscle proliferation and differentiation (myomiRs) e.g., miR-1, 133a/b, 206, 208a/b, 499a/b and others are more and more coming to limelight, but except for miR-1, these were not associated to the over- and down-expressed VL/SOL proteins (genes) experimentally detected by Moriggi et al. (2010). Moreover, we find some miRs that were not described by others. For examples, in murine gastrocnemius in spaceflight environment the ratio of miR-1/ miR-133a was significantly increased (Allen et al., 2009) but none of ours were found. These differences might be accounted for different models (animals vs. human), different environment (spaceflight vs. bed rest), different target (gastrocnemius vs. VL and SOL).

## Conclusion

In conclusion, our bioinformatics analysis revealed that conflicting signals are taking place simultaneously in reduced gravity environment, resulting in impairment between proliferation and differentiation signaling pathways which might be responsible for the observed atrophy (Figure 1). The same predicted dysregulated miRs are, at the same time, also responsible for an inflammation response, which might be the reason of atrophy outcome thus suggesting that immune dysregulation is one of the molecular pathways ensuing microgravity-generated muscle atrophy. In this report we highlighted the roles played by the pathways controlled mainly by let-7a and miR-1, miR-125b-5p, miR-95-5p, miR-222-3p. The miRs here discussed are, of course, not exhaustive of the mechanisms involved in skeletal muscle response to microgravity, however, they highlight the important role played by inflammation in muscle atrophy as already demonstrated in other forms of skeletal muscle pathologies.

## Author Contributions

LT and MCA made the bioinformatic analysis. LT, MCA, and AC wrote the paper. LC revised the manuscript.

## Conflict of Interest Statement

The authors declare that the research was conducted in the absence of any commercial or financial relationships that could be construed as a potential conflict of interest.

## Footnotes

1 http://www.mirnet.ca

2 https://www.ncbi.nlm.nih.gov/pubmed
